# The Financial Burden of Delayed Hip Fracture Surgery: A Single-Center Experience

**DOI:** 10.7759/cureus.13952

**Published:** 2021-03-17

**Authors:** Vasiliki Chatziravdeli, Angelo V Vasiliadis, Polychronis Vazakidis, Maria Tsatlidou, George N Katsaras, Anastasios Beletsiotis

**Affiliations:** 1 2nd Orthopaedic Department, General Hospital of Thessaloniki “Papageorgiou”, Thessaloniki, GRC; 2 Paediatric Orthopaedics, University Hospital of Southampton, Southampton, GBR; 3 2nd Orthopaedic Department, General Hospital of Thessaloniki "Papageorgiou", Thessaloniki, GRC; 4 Paediatrics, General Hospital of Pella (Hospital Unit of Edessa), Edessa, GRC

**Keywords:** financial, hip fracture, fragility, delayed surgery, cost, hospitalisation

## Abstract

Fragility hip fractures have become a worldwide epidemic with serious socioeconomic implications. The projected number of hip fractures by 2050 is estimated to reach 4.5 million cases. The aim of this study was to calculate the in-hospital financial burden on public health insurance funds related to the delayed treatment of hip fractures. This research took place in a tertiary university hospital that is a major trauma center in Thessaloniki, which is second largest city in Greece . A retrospective search was conducted in the electronic hospital database for patients older than 65 years, with low energy hip fractures that were surgically treated between November 18, 2018, and October 20, 2019. Age, length of stay (LOS), days to surgery, postoperative LOS, anticoagulation medication, major and minor complications, and the reimbursement that the hospital received from public health insurance funds were recorded. Cost deviation from the standard tariff for the treatment of these fractures was also calculated. Of a total of 145 patients, 32.4% had early surgery as opposed to 67.6% who were operated after 48 hours from admission. The excess financial burden from the baseline reimbursement for those operated within 48 hours from admission was 4,074.64€, while for the group that received delayed surgery it was 45,654.14€. Patients under any form of anticoagulation therapy were seven times more probable to have delayed surgery [OR=6.8; 95% confidence interval (CI): 2.97-18.18; p<0.01] and were 3.5 times more probable to have minor complications (OR: 3.6; 95% CI: 1.19-11.23; p<0.017). Early surgery is beneficial to the patient and reduces the economic burden on healthcare public funds. Every effort should be made to manage these patients in a timely manner.

## Introduction

Fragility fractures in the ageing population, and among them hip fractures, are considered a worldwide epidemic and are a leading cause of concern in a number of countries because of the serious socioeconomic implications as well as the burden on public health care systems. There is a great variation in the incidence among countries, with northern European countries like Denmark, Sweden, Norway, and Austria having the highest annual age-standardized incidence compared to Tunisia and Equador having the lowest [1]. Hip fracture incidence can vary as much as 200-fold and 140-fold in women and men, respectively, between countries [2], and women are more likely to suffer a hip fracture than men, with an average ratio of 2:1 [3]. In the five largest European countries plus Sweden, 526,470 hip fractures occurred in 2017, and the projection of the number of hip fractures globally by 2050 is estimated to reach 4.5 million [4,5]. In-hospital treatment cost for this condition is significant, arising from the duration of hospitalization, laboratory and radiology testing, surgical fracture stabilization with the use of implants, and drug administration, as well as rehabilitation programs that commence postoperatively and continue thereafter. Due to the fact that the population suffering from hip fractures is mainly geriatric, concomitant comorbidities can further increase the cost of inpatient treatment because of the need for preoperative patient optimization and the increased probability of complications [6-9].

The primary aim of this study was to calculate the in-hospital financial burden on public health insurance funds related to the delayed treatment of hip fractures during a one-year period in a tertiary center. The secondary aim was to determine the predisposing factors influencing time to surgical management and the concomitant factors that contribute to increased cost of hospitalization.

## Materials and methods

A retrospective search was conducted in a single-center major trauma academic hospital. The hospital's electronic database was used to identify patients with hip fractures that were surgically treated between November 18, 2018, and October 20, 2019. The search criteria utilized the codes S72.0 and S72.1 according to the International Statistical Classification of Diseases and Related Health Problems 10th Revision (ICD-10) [10], which account for femoral neck and intertrochanteric fractures, respectively. The study included patients who were older than 65 years of age and suffered low energy hip fractures treated with surgical fracture stabilization. Patients younger than 65 years with high energy femoral neck or peritrochanteric fractures and femoral shaft fractures were excluded. Furthermore, patients who were treated conservatively and those deceased before receiving surgical treatment were excluded as well. Surgical treatment for intertrochanteric fractures consisted of cephalomedullary nailing, and femoral neck fractures were treated with hip hemiarthroplasty. Age, length of stay (LOS), days to surgery (DTS), postoperative LOS (PLOS), anticoagulation medication, major and minor complications, and the reimbursement that the hospital received from public health insurance funds were recorded. According to the Diagnosis-Related Group (DRG) [11] that apply in our country and the information provided from the Hospital's Finance & Account Office, an average of 2,765 Euros (€) is refunded for the treatment of patients with hip fractures and cover for nine hospitalization days. The cost deviation from this amount was also calculated for each patient during the study period, with the aid of the Hospital’s Account and Finance Department. Minor complications were wound drainage treated without the need of surgery and urinary tract infection, whereas major complications were respiratory infection, myocardial and cerebrovascular adverse events, nerve palsies, and death. The data were analyzed using SPSS Statistical Package 26 (IBM Corp., Armonk, NY, USA). For comparisons of categorical variables, the Pearson chi-square test was used, and for continuous variables, the Mann-Whitney U test for independent samples was used. Logistic regression was used to determine factors that influence timing of surgery. Surgery performed after 48 hours from admission was considered as a delayed treatment. This study received Institutional Board approval.

## Results

During the study period, a total of 145 patients over 65 years old (mean: 82.7) were surgically treated in our institution for low-energy hip fracture. Of the 145 patients, 71 suffered a femoral neck fracture and 74 from an intertrochanteric fracture. The total cost of treatment was calculated as 447,904.6€, and the total additional cost from the baseline price was 49,728.78€. Of the 145 patients, 53 were hospitalized for less than nine days and 92 were inpatients for more than nine days, and the additional cost, according to the DRG, was 5,600.2€ for the first group and 44,128.58€ for the second, which was statistically significant (p<0.01). Only 47 (32.4%) patients were operated within 48 hours, and 98 patients (67.6%) had delayed surgery. The median duration of hospitalization was seven days (IQR: 6-7.25) and 12 days (IQR: 11-15) for each group, respectively, which was statistically significant (p<0.01). Of the people treated with early surgery, 91.5% had nine or less hospitalization days compared to only 11.2% in the delayed surgery group. There was no difference concerning delayed surgery between those who suffered from femoral neck fracture and those who suffered from intertrochanteric fracture (p=0.4), with the majority of cases operated after 48 hours in both groups.

The excess financial burden from the baseline reimbursement for those operated within 48 hours from admission was 4,074.64€, while for the group that received delayed surgery it was 45,654.14€ (Table 1).

**Table 1:** Demographics of early versus delayed surgery NOF, femoral neck fracture; INTR, intertrochanteric fracture; IQR, interquartile range

No. of patients (total = 145)	Early surgery, 47 (32.4%)	Delayed surgery, 98 (67.6%)	p-Value
NOF (total = 71)	25 (35.2%)	46 (64.8%)	
INTR (total = 74)	21 (28.7%)	52 (71.3%)	0.4
Days of hospitalization, median (IQR)	7 (6-7.25)	12 (11-15)	0.01
Excess cost	4,074€	45,654.14€	0.01
Total cost (median)	131,013€ (2,777)	319,655€ (3,031)	0.01

Statistical analysis showed that patients under any form of anticoagulation therapy were seven times more probable to have delayed surgery (OR=6.8; 95% confidence interval (CI): 2.97-18.18; p<0.01).

Those who did not receive any form of anticoagulation were 4.4 times more probable to be hospitalized for less than nine days compared to patients under anticoagulation (OR: 4.4; 95% CI: 2.12-9.36; p<0.01). Another parameter that was correlated with delayed surgery was the frequency of minor complications. Patients having delayed surgery had 3.5 times greater probability to have minor complications (OR: 3.6; 95% CI: 1.19-11.23; p<0.017), whereas major complications were not influenced by delay in surgery (Table 2).

**Table 2:** Factors associated with delayed hip fracture surgery OR, odds ratio; CI, confidence interval

Delayed surgery	OR (95% CI)	p-Value
Anticoagulation	6.8 (2.97-18.18)	0.01
Minor complications	3.6 (1.19-11.23)	0.017

## Discussion

Fragility hip fractures are more common in geriatric patients who often receive medication, such as anticoagulants, for concomitant conditions such as cardiovascular and/or cerebrovascular disease. In a recent study, it was estimated that 30% of people presenting with hip fracture receive anticoagulation [12].

This can have a great impact on the timing of surgery, as reversal of the anticoagulation effect is often necessary for performing regional anesthesia, diminishing bleeding diathesis, and, in some cases, patient optimization as well [13]. Time to surgery is also influenced by other factors such as operating theater capacity, patient optimization, and day of admission. Even in countries like the UK, were the “Best Practice Tariff” (extra economic benefit per patient) is implemented, in 2017, 30% of patients were operated after 36 hours from presentation [13,14]. In our study, factors that influenced the cost of hip fracture treatment were LOS and the existence of major and minor complications. In our study, patients taking anticoagulation therapy had a significant risk of delayed surgery, which prolonged LOS and increased the probability of minor complications. In a recent study by Cordero et al., it was demonstrated that a 24-hour delay in surgery posed a significant risk of wound infection [15]. This conclusion is in accordance with our findings, where minor complications, mainly wound drainage, were also correlated with delayed surgery. Therefore, anticoagulation treatment was an indirect factor that influenced the cost of treatment for patients with hip fractures.

A recent study evaluating the factors that influence the money expenditure for treating patients with hip fractures showed that ward costs comprised 84% of expenditure, thus demonstrating the economic burden placed on health care systems by prolonged hospitalization [6].

Delay of more than 48 hours in the management of hip fractures is a risk factor for major complications such as pneumonia and pulmonary edema, with consequent prolongation in the length of hospitalization and higher mortality and morbidity rates [16]. As demonstrated in our study, major complications constitute a risk factor that increases the cost of treatment, even in cases without prolonged LOS. A possible reason could be the fact that major complications require interventions that are often costly, such as intensive care unit admission, expensive drug therapy, and invasive procedures. Knauf et al. in a prospective study demonstrated that all types of severe complications, increased the inpatient cost. Adverse events such as myocardial infarction, stroke, and acute renal failure requiring dialysis were the most costly, followed closely by complications that necessitated surgical revision. The latter also had the highest one-year mortality rate among the other types of major complications [17].

Our research indicates that there was a significantly higher percentage of patients who were operated after 48 hours. Only 32.4% were treated within 48 hours, which is the recommended time frame in order to achieve optimal results for the patients and diminish complication rates [16].

Patients on anticoagulants experienced the most prominent delays, with a mean time to surgery of 6.06 days (95% CI: 5.35-6.76) and had also greater probability for complications. There is a lack of guidelines from the International Orthopaedic Association to guide surgeons regarding the management of patients under anticoagulation that present with hip fracture [13]. Orthopedic surgeons rely mostly on guidelines provided by other International Medical Bodies, such as the European Heart Rhythm Association and the European Society of Anaesthesiology, that have published guidelines regarding time from discontinuation of anticoagulant to surgery. They advise on a 24-hour time lapse from the last dose until urgent surgery, for patients under NOACs (newer oral anticoagulants), while this time frame increases if a neuraxial block is to be performed, from three to five days, depending on the type of NOAC and renal function [18,19]. Clopidogrel and newer antiplatelets need to be discontinued five to seven days in order to perform regional anesthesia, but there is no contraindication for early surgery in terms of substantial increase in perioperative bleeding and mortality. A review and meta-analysis including patients on clopidogrel who had early surgery demonstrated only a marginal increase in transfusion rates and no significant difference in mortality [20]. For warfarin, the proposed practice is reversal of anticoagulation effect in order to perform early surgery, and aspirin is no longer a problem because it does not have to be discontinued [19,21]. In light of the above, our mean time to surgery for anticoagulated patients was significantly higher than the recommended practice. This could be attributed to a number of reasons, such as longer waiting periods from discontinuation of the anticoagulant until surgery, no use of agents for reversal of anticoagulation, diminished operating theater capacity, and shortage of medical/nursing staff. In the last decade, our country has suffered under a continued economic recession that resulted in cut backs in hospital funding and medical and nursing staff, thus creating great difficulties in managing patients who need urgent treatment. Furthermore, due to the fact that our hospital is a tertiary hospital, life-threatening and more urgent surgery of other surgical specialties take priority over hip fracture surgery, and, combined with lack of personnel, this results in even greater delays.

Limitations of this study is that it is a retrospective observational study performed in a single center. Subgroup analysis according to type of anticoagulant was not performed because of the limited number of cases.

## Conclusions

Hip fracture is an orthopedic urgency and should be managed according to the international recommendations. Early surgery is beneficial to the patient in terms of early mobilization and better functional outcome and reduces complication rates as well as economic burden on healthcare public funds. Every effort should be made to manage these patients in a timely manner and preferably in less than 48 hours, which is in the best interest of patients and public health care systems.
